# Effect of thermocycling and varying polymerization techniques on the restorative interface of class V cavities restored with different composite resin systems

**DOI:** 10.4317/jced.53481

**Published:** 2017-03-01

**Authors:** Jefferson-Ricardo Pereira, Lindomar-Corrêa Júnior, Marcus-Vinicius-Reis Só, Newton-Fahl Júnior

**Affiliations:** 1 DDS, MSc, PhD, Department of Prosthodontics, University of Southern Santa Catarina, Tubarão, Santa Catarina, Brazil; 2 DDS, Department of Prosthodontics, University of Southern Santa Catarina, Tubarão, Santa Catarina, Brazil; 3 DDS, MSc, PhD, Department of Conservative Dentistry, Rio Grande do Sul Federal University, Porto Alegre, RS, Brazil; 4 DDS, MSc, Newton Fahl Center, Curitiba, PR, Brazil

## Abstract

**Background:**

To evaluate marginal microleakage of two composite resins - a methacrylate- and a silorane-based submitted to different polymerization techniques and thermocycling.

**Material and Methods:**

Ninety-six class V cavities were prepared in sound human molars and restored under different polymerization and thermocycling regimens. The adhesive systems employed were Adper Scotchbond Multipurpose and Filtek P-90 for cavities restored with Z250 and P-90. The specimens were restored with Z250 or P-90, and divided into 3 subgroups with different polymerization techniques. The data were analyzed by Three way Analysis of Variance Test (*p*<0.05).

**Results:**

Micro infiltration lower scores were found in groups which were used silorane-based resin with significant statistical difference compared with the specimens restored with methacrylate-based resin, independently of polymerization type used and thermocycling (*P*>0.001).

**Conclusions:**

Silorane-based composite resins present lower marginal microleakage values when compared to methacrylate-based composites resins.

** Key words:**Composite resin, microleakage, polymerization.

## Introduction

Dental composites were developed as an esthetic alternative to the long-used amalgam-based restorative materials and have become more popular and widely used in current clinical dentistry (1,2). Benefits incurring from the use of composite resins in lieu of amalgam include the mimicry of natural tooth color, absence of mercury, low thermal conductibility, and the ability to bond to dental structures through adhesive systems (3).

Polymerization is the chemical reaction that converts small molecules, the monomers, into large chains called polymers (4). Light intensity and exposure time have been shown to be key factors in determining the speed at which free radicals are released to initiate the polymerization reaction and the extent of cure of composite resins, respectively (5). In this sense, manufacturers have recommended high intensity lights to process a higher conversion degree of monomers into polymers, thus improving the mechanical properties of composite resins. However, conversion degree is always associated with an inconvenience of great clinical relevance: polymerization shrinkage (6,7).

The complete polymerization shrinkage can be divided in pre and post-gel phases. During pre-gel polymerization, flows and tensions inside of material structure are released; during the post-gel phase, however, there is significant stress induction at tooth/restoration interface (8). Because of this, several authors have suggested methods of polymerization that delay pre-gel time, in order to have a slower rate of monomer conversion, which, in turn, allows for a better resin flow with decreased contraction stress. Unlike conventional polymerization, whereby the light intensity is fixed, an initial photoactivation of low light intensity followed by high light intensity final photoactivation, would tend to minimize marginal cracks, without compromising the physical and mechanical properties of composite resins. This process is termed Soft-Start polymerization (9).

Shrinkage generates important clinical drawback, such as gaps between tooth and restoration (10,11). The occurrence of these gaps is due to the forces generated in the material body, which are transmitted to the tooth/restoration interface. It compromises the bond strength and marginal integrity. The restoration becomes consequently more susceptible to infiltration, secondary caries and postoperative sensitivity (12).

Recent research in the field of dental materials introduced silorane-based composite resins in substitution of methacrylate-based composite resins (13). This material is derivative of oxirane and siloxane molecules, which polymerize themselves with an opening of cationic ring, in maximum overcoming to the clinical inconvenience of material polymerization shrinkage related to methacrylate-based composite resins (14). Furthermore, this material has shown good mechanical properties (15) and fluid stability in oral cavities simulators (16), which encourages its clinical use.

In light of the above, the objective of this study was to evaluate the effect of thermocycling and of varying polymerization techniques on the restored surfaces of Class V cavities with different composite resins. The hypothesis of this study was that there would be significant differences between the composite resins, and among the intensity polymerization, and thermocycling variables.

## Material and Methods

This study was approved by Unisul ethics comitte. Forty-eight sound human molars were selected from a Teeth Bank of the University of Southern Santa Catarina. The teeth had been recently extracted by periodontal or orthodontic indications and stored in 10% formalin solution, in order to maintain their integrity (17).

Teeth were cleaned with water-pumice slurry in a prophylaxis rubber cup and rinsed with copious water before preparation of the cavities. All the teeth were carefully observed through a 4 X binocular loupe (Bio Art Equipamentos Odontológicos Ltda, São Carlos-SP, Brazil) in order to appraise the absence of cracks, deterioration, fissures, or previous restorations, which could compromise final results.

Class V cavities were prepared on the buccal and lingual aspects of all teeth. The cavities dimensions were 3mm mesio-distally, 3mm cervico-occlusally and 3mm deep. The cavities were performed using a cylindrical diamond bur with rounded end (#2143, KG Sorensen, São Paulo, SP, Brazil) at high speed and under air/water cooling with a cursor that limits the drill action to a pre-determined depth. Finishing of the cavity preparations was carried out also under water cooling using fine-gritted diamond burs of the same diameter, (#2143 F – KG Sorensen, São Paulo, SP, Brazil). The dimensions of the cavities were verified using a precision digital caliper (Digimatic, 500-181U; Mitutoyo Corp, Tokio, Japan). Between each stage of this experiment, teeth were stored in 37ºC distilled water. All 48 teeth were randomly divided in 12 experimental groups. Six groups were restored using a methacrylate-based resin and a total etch three-step adhesive system (Z) and six of them using a silorane-based restorative mate-rial and a self-etch one-step adhesive system developed for silorane-based restorative system (P), these groups were divided into 3 subgroups and subsequently subjected to high (H), soft-start (S), and low intensity polymerization (L). Considering similar groups, one was submitted to thermocycling (T) while the other to no thermocycling (N) ( Table 1).

**Table 1:**
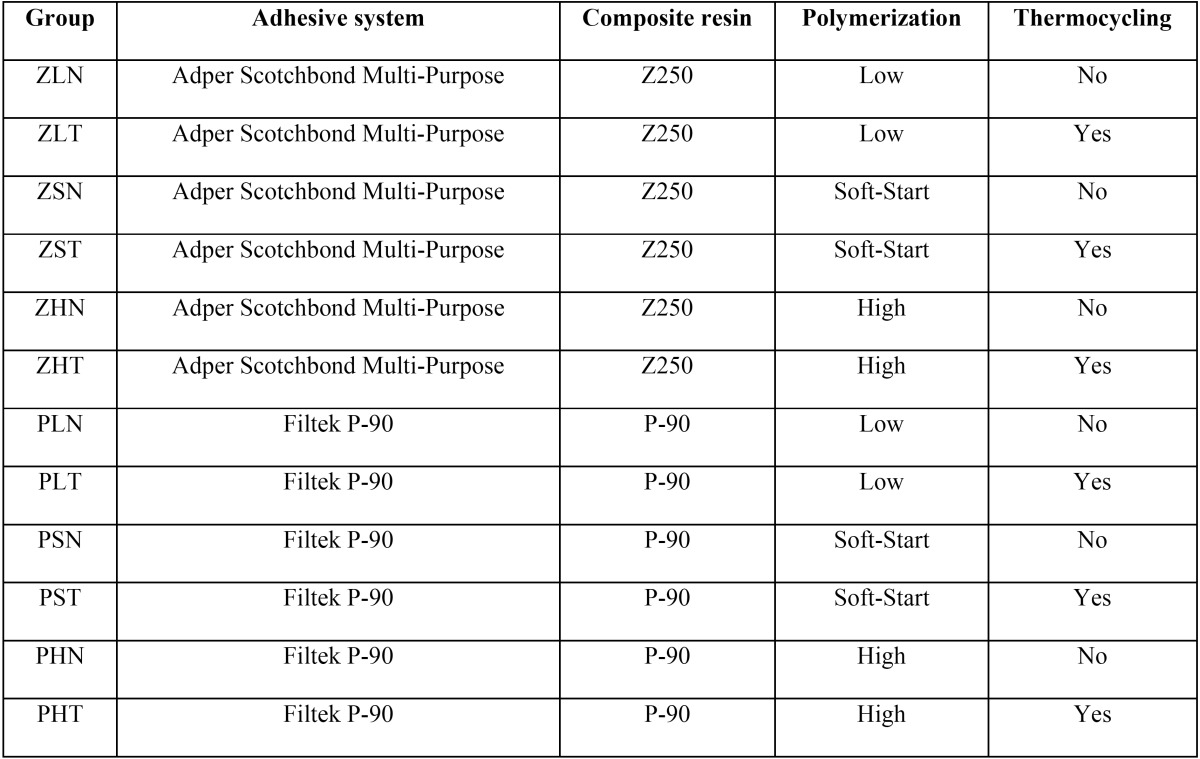
The groups presented in this study. All of material were from 3M ESPE, USA.

For the groups restored with Z250, after prepare the cavity, the cavities were etched during 15 seconds and it was washed with air spray and water during de 10 seconds. Humidity excess was dried with cotton pellet without air drying. The Scotchbond Multi-purpose primer was applied with a microbrush, the excess was suctioned with an endodontic suction tip, and, after 5 minutes, the Scotchbond Multipurpose bond agent was applied with a microbrush in only one layer, the excess was suctioned with an endodontic suction tip and light-cured for 10 seconds with a curing unit (Blue Fase, Ivoclar Vivadent) using a 9mm-diameter light guide and keeping a distance between the light guide surface and the specimens near 1 mm.

For the groups restored with P-90 the dentin was not etched. The methodology to use silorane adhesive system was exactly the same using scothbond system.

Composite resins were applied in the different groups using the incremental technique by insertion of three increments: two of them oblique and one covering both previous increments. In the groups ZLN, PLN, ZLT and PLT, the photo activation of each increment was performed by the technique Low with fixed light intensity at 650mW/cm2 for 20 s; in the groups ZSN, PSN, ZST and PST, the photo activation in each increment was performed by Soft-Start technique at starting in 0mW/cm2 to 650mW/cm2 in 5 s + 1200mW/cm2 for 20 s; in the groups ZHN, PHN, ZHT and PHT, the photo activation in each increment was performed by the technique High in 1200mW/cm2. All of them using the curing unit Blue Fase (Ivoclar-Vivadent). Restorations were finalized using finishing burs (KG Sorensen, São Paulo, SP, Brazil) in order to remove gross excess, followed by sof-lex disc (3M ESPE).

After finishing the specimens from groups ZLT, PLT, ZST, PST, ZHT, and PHT, they were taken to the thermocycling and submitted to 5000 cycles during 30 seconds in 5º and 55ºC temperature, while specimens from groups ZLN, PLN, ZSN, PSN, ZHN, and PHN stayed immersed in 37ºC distilled water.

Specimens were embedded in 1% methylene blue (Prolabo, Paris, France) during 48 hours. The evaluation of pigment penetration in the interfaces was performed after specimens were washed in distilled water. Microleakage analysis was performed under binocular microscope (X10, model S2H, Olympus corp., Tokyo, Japan) after each crown was cross-sectioned buccolingually using a diamond saw (Isomet 1000, Buehler, Lake Bluff, Ill, USA) at 400 rpm and 250 g, under copious water-cooling.

Evaluation criteria consisted in scores from 0 to 4 which mean: score 0- absence of infiltration; 1- Infiltration until the half of surrounding wall; 2- Infiltration in all surrounding wall; 3- Infiltration in surrounding wall and axial; 4- Infiltration in surrounding wall and axial towards the pulp. The results founded were statistically analyzed by Three way Analysis of Variance Test (*p*<0.05).

## Results

 Table 2 shows microleakage scores found in different groups, which were combined composite resins materials, polymerization systems, and thermocycling.  Table 3 shows microleakage scores found considering separately composite resins materials, polymerization systems, and thermocycling.

**Table 2:**
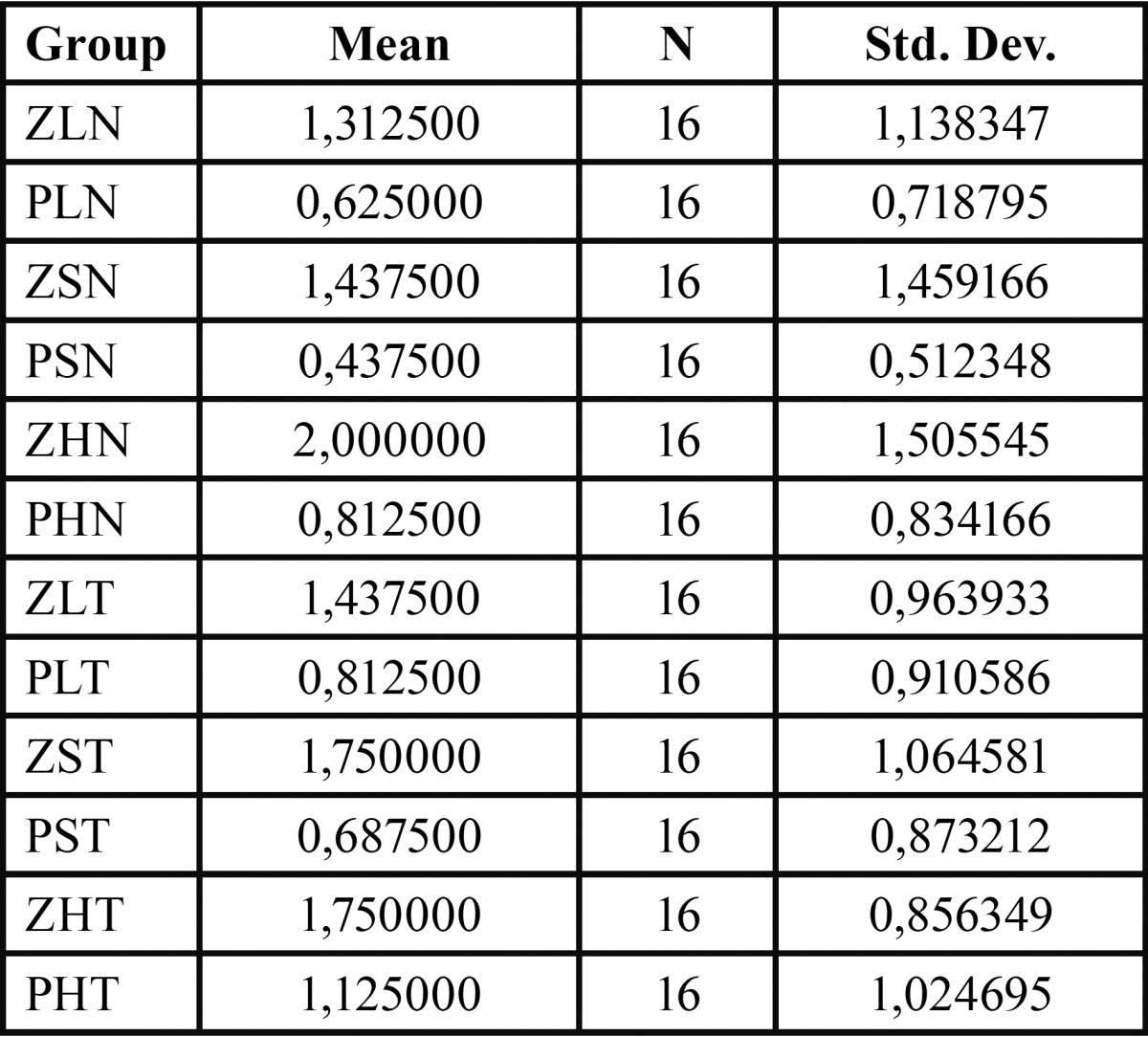
Microleakage scores found in different groups which were combined composite resins materials, polymerization systems, and thermocycling.

**Table 3:**
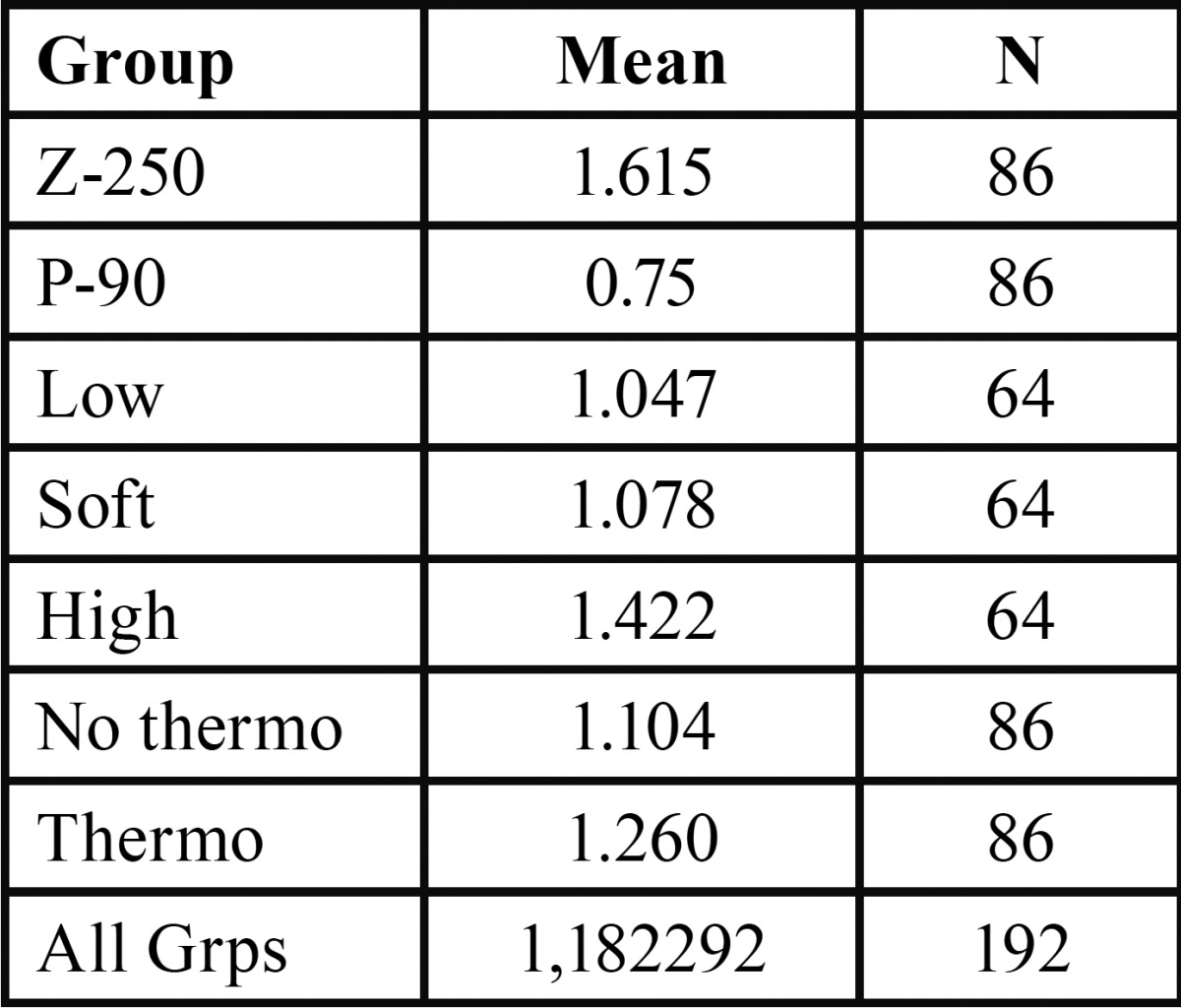
Microleakage scores found in different groups considering separately composite resins materials, polymerization systems, and thermocycling.

It was possible verify that micro infiltration lower scores were found in groups which were used silorane-based resin with significant statistical difference compared with the specimens restored with methacrylate-based resin, independently of polymerization type used and thermocycling (*p*>0.001). For other groups there were no statistical significant differences among them.

Microleakage scores were submitted to statistical analysis by three way Analysis of Variance Test in order to verify the existence of difference among tested groups ( Table 4).

**Table 4:**
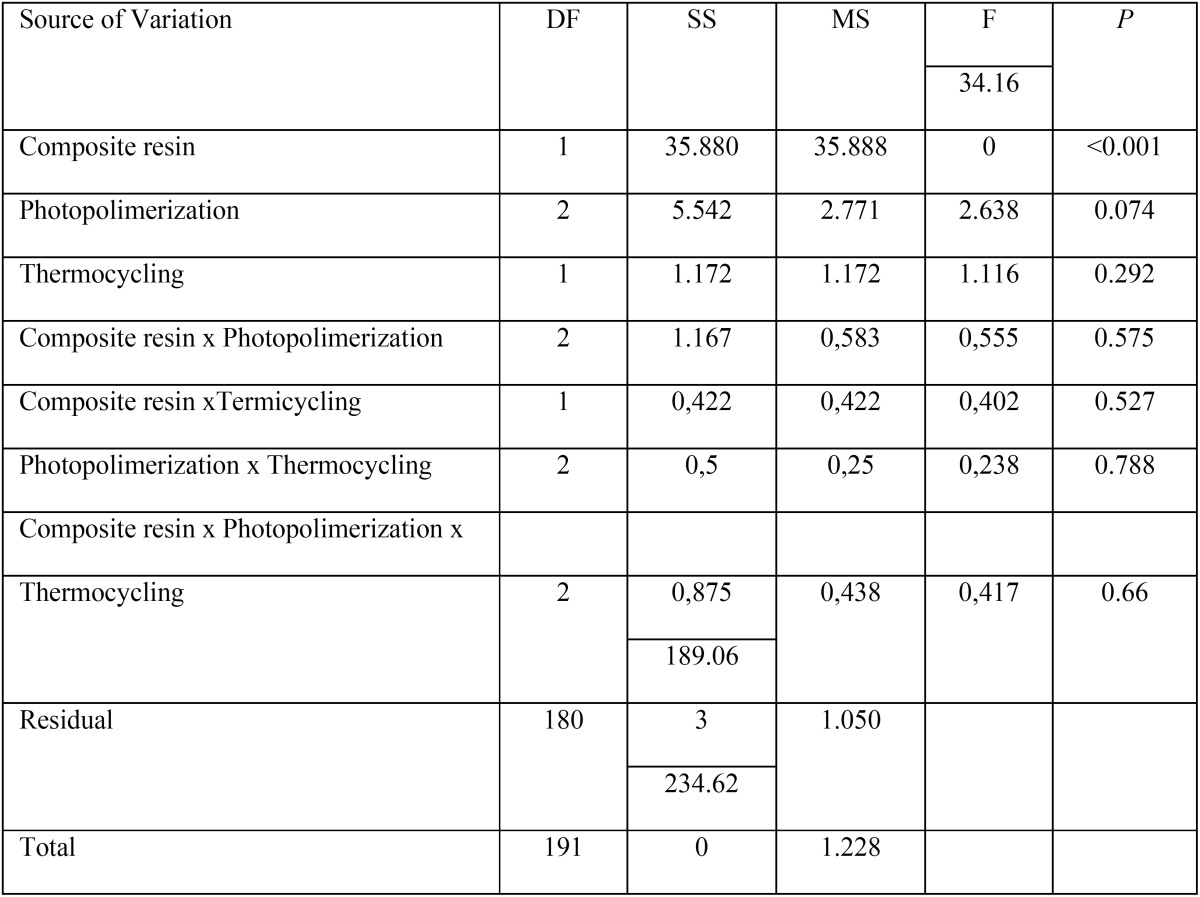
Three Way Analysis of Variance.

## Discussion

The results of this study accepted the hypothesis that there were significant differences between the composite resins systems, polymerization system, and thermocycling. It was possible to observe that significant lower microleakage scores were found in groups restored with silorane based resin and it was no found significant differences comparing the different groups according in thermocycling and polymerization. This fact can be explained by polymerization kinetics of silorane that can be slower and show the property of cationic ring opening able to reduce its contraction to levels lower than 1% volume (14,18).

Even thought, Filtek silorane presents a filler load of 53% by volume whereas Z-250 presents a filler load of 60%. This might provide a better flow of silorane-based materials, which develop lower stress during polymerization and, consequently, better marginal integrity maintenance when compared to methacrylate-based composites. This fact could be observed in this study when comparing the different composite resins.

The results of this study showed that there is no statistical difference among groups with or without thermocycling, even in silorane-based composite resin or methacrylate-based composite resin. These results are in accordance with Eakle (19) who found that there is no difference in microleakage when the samples are submitted or not to the thermal challenge. According to Veronezi *et al.* (20), the difference in thermal expansion between the material and the dental structure provokes alternating increase and decrease of space between material and dental structure, which can provide an increase in marginal microleakage in composite resin restoration. The incremental technique may be reduced the contraction and consequently the microleakage of all restoration.

Another important factor that may have influenced the higher microleakage was the high intensity polymerization regimen, as it provides a prolonged duration of light exposure under high intensity. According to some authors, this light activation method is ideal for polymerizing composite resins because it provides adequate polymerization depth with improved physical and mechanical properties (21). On the other hand, some authors assert that this type of polymerization may not prolong pre-gel stage, which is responsible for a great part of compensation of polymerization contraction. In this way, crosslinks are rapidly established and the flow ability of resin and every contraction stress generated is transferred to the bond interface tooth/restoration. It can compromise the marginal integrity and increase microleakage levels (22). These finds could not be observed in the present study and can be explained by the use of the incremental technique.

Despite the influence of thermocycling factors and polymerization methods, this study highlights the chemical composition of material above other factors. Even with the possibility of thermal influence and of current different polymerization methods that prolong the pre-gel stage, this study did not evidence the influence of these factors in groups tested with either silorane-based or with metacrylate-based composite resins. In these groups there is no statistically significant differences, which allows the inference as more important factor in marginal microleakage decrease being the chemical composition of silorane and its ability of opening in the moment of polymerization.

## Conclusions

Considering the results of this study, it is possible conclude that silorane presents low values of marginal microleakage when compared to methacrylate-based composites. These results seem to be associated to the chemical composition inherent to the material, instead of to different polymerization systems and to the thermal challenge promoted by thermo cycling.
